# Early-phase ^18^F-FP-CIT and ^18^F-flutemetamol PET were significantly correlated

**DOI:** 10.1038/s41598-021-91891-z

**Published:** 2021-06-10

**Authors:** Young-Sil An, Jung Han Yoon, Sang Joon Son, Chang Hyung Hong, Su Jin Lee, Joon-Kee Yoon

**Affiliations:** 1 Department of Nuclear Medicine and Molecular Imaging, School of Medicine, Ajou University, 206, World cup-ro, Yeongtong-gu, Suwon-si, 16499 Gyeonggi-do Korea; 2 Department of Neurology, Ajou University School of Medicine, Suwon, Korea; 3 Department of Psychiatry, Ajou University School of Medicine, Suwon, Korea

**Keywords:** Molecular medicine, Neurology

## Abstract

Little is known about whether early-phase PET images of ^18^F-FP-CIT match those of amyloid PET. Here, we compared early-phase ^18^F-FP-CIT and ^18^F-flutemetamol PET images in patients who underwent both within a 1-month interval. The SUVR on early-phase ^18^F-FP-CIT PET (median, 0.86) was significantly lower than that of ^18^F-flutemetamol PET (median, 0.91, *p* < 0.001) for total brain regions including all cerebral lobes and central structures. This significant difference persisted for each brain region except central structures (*p* = 0.232). The SUVR of total brain regions obtained from early ^18^F-FP-CIT PET showed a very strong correlation with that of ^18^F-flutemetamol PET (*rho* = 0.80, *p* < 0.001). Among the kinetic parameters, only *R*1 showed a statistically significant correlation between the two techniques for all brain regions (*rho* = 0.89, *p* < 0.001). *R*1 from ^18^F-FP-CIT (median, 0.77) was significantly lower in all areas of the brain compared to *R*1 from ^18^F-flutemetamol PET (median, 0.81, *p* < 0.001).^18^F-FP-CIT demonstrated lower uptake in cortical brain regions than ^18^F-flutemetamol on early-phase PET. However, both early-phase PETs demonstrated significant correlation of uptake.

## Introduction

Parkinson's disease (PD) and Alzheimer's dementia (AD) are representative neurodegenerative diseases, and the number of patients afflicted is rapidly increasing in aging societies^1,2^. In the field of positron emission tomography (PET), dopamine transporter PET and amyloid PET are widely used in clinical practice to evaluate PD^3,4^ and for differential diagnosis of AD^5^, respectively. In addition, brain perfusion imaging could provide complementary information when evaluating these patients^6–8^. However, oxygen-15-labeled water for cerebral perfusion PET image has a short half-life (2.04 min), this technique is limited to institutions that have a cyclotron. Although brain perfusion single photon emission computed tomography using ^99m^Tc-ethyl cysteinate dimer or ^99m^Tc-hexamethylpropylene amine oxime exists, it offers lower resolution than PET^9^. ^18^F-fluorodeoxyglucose (FDG) brain PET could be used based on the fact that brain blood flow and glucose metabolism are well coupled^10,11^, but it also has limitations in that dual-biomarker positron PET can lead to increased costs, radiation exposure, longer scanning time and patient discomfort^12^.

To address these issues, dual-phase imaging has been attempted with *N*-(3-fluoropropyl)-2β carboxymethoxy-3β-(4-iodophenyl) nortropane (^18^F-FP-CIT) and amyloid PET, and the usefulness of this approach has been demonstrated by several previous studies^13–17^. In the dual-phase protocol, early-phase images taken 10–15 min after injection of the radiopharmaceutical are obtained in addition to the usual delayed image. This method is used under the assumption that the early-phase images can reflect brain perfusion^13,14,18^. Since the dual-phase protocol is advantageous in that two functional images can be obtained with a single injection of radiopharmaceuticals, many institutions obtain an additional early-phase scan during routine ^18^F-FP-CIT or amyloid PET. Whether early-phase images from ^18^F-FP-CIT and amyloid PET would exhibit similar uptake remains unclear. Assuming that early-phase PET images commonly reflect perfusion, both early PET images would have to be closely matched. However, no previous studies have directly compared early-phase uptake between these techniques.

The aim of this study was to investigate whether ^18^F-FP-CIT uptake in the early phase correlated with early amyloid PET, and whether there were any differences between these techniques.

## Results

### Early-phase standardized uptake value ratios (SUVRs) from ^18^F-FP-CIT and ^18^F-flutemetamol PET

**Figure 1:**
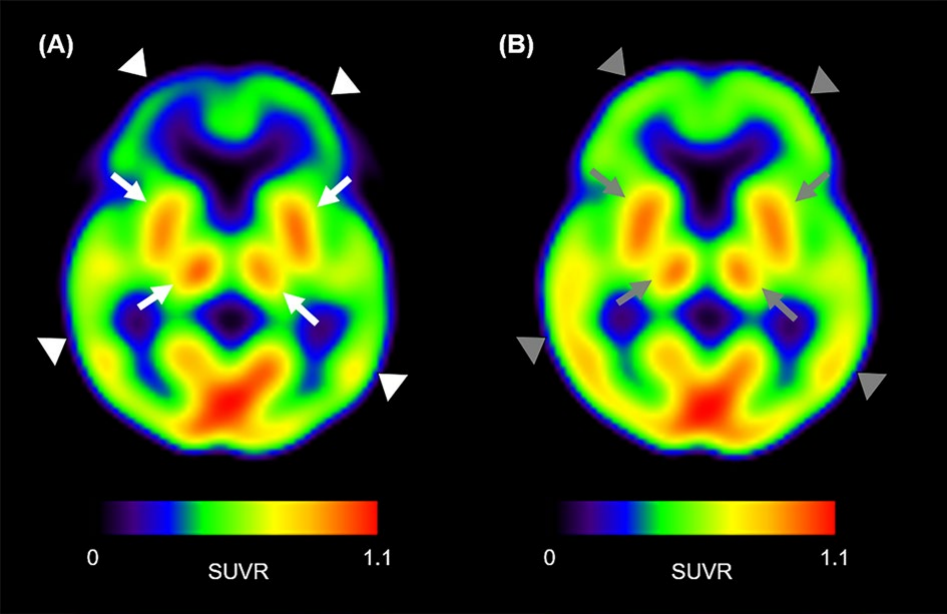
Representative images of early-phase PETs. The ^18^F-FP-CIT SUVR image showed less uptake in cortical areas (white arrowheads in **A**) than the ^18^F-flutemetamol SUVR image (grey arrowheads in **B**), while the central structures showed similar activity in the two PETs (white arrows in **A** and grey arrows in **B**).

The SUVR obtained from ^18^F-FP-CIT PET (median [interquartile range (IQR)], 0.86 [0.79 to 0.89]) was significantly lower than that obtained from ^18^F-flutemetamol PET (0.91 [0.85–0.95], *p* < 0.001) for overall total brain regions including all cerebral lobes and central structures. In the results for each brain area, the SUVR from ^18^F-FP-CIT PET for cortical brain regions (i.e., frontal, occipital, parietal and temporal lobes) showed a significantly lower value than ^18^F-flutemetamol PET (all *p* < 0.05), with the exception of the central structures (*p* = 0.232). The detailed results for SUVR are presented in Table 1 and representative images that support these results are shown in Fig. 1.

**Table 1:** Early-phase parameters of ^18^F-FP-CIT PET and ^18^F-flutemetamol PET.

	SUVR (median, IQR*)			*R*1 (median, IQR*)		
	^18^F-FP-CIT PET	^18^F-flutemetamol PET	*p*-value^†^	^18^F-FP-CIT PET	^18^F-flutemetamol PET	*p*-value^†^
Central structures (*n* = 10)	0.83 (0.78–0.96)	0.88 (0.84–0.93)	0.232	0.63 (0.61–0.68)	0.69 (0.67–0.75)	0.027
Frontal lobe (*n* = 10)	0.86 (0.82–0.90)	0.91 (0.88–0.95)	0.002	0.81 (0.80–0.84)	0.88 (0.82–0.91)	0.004
Occipital lobe (*n* = 10)	0.89 (0.87–0.95)	0.99 (0.95–1.02)	0.002	0.82 (0.80–0.90)	0.94 (0.91–0.00)	0.004
Parietal lobe (*n* = 10)	0.83 (0.80–0.87)	0.92 (0.86–0.95)	0.002	0.78 (0.71–0.87)	0.84 (0.78–0.91)	0.014
Temporal lobe (*n* = 10)	0.79 (0.78–0.82)	0.83 (0.82–0.85)	0.016	0.71 (0.67–0.77)	0.75 (0.73–0.78)	0.019
Total brain regions^‡^ (*n* = 50)	0.86 (0.82–0.87)	0.91 (0.85–0.95)	< 0.001	0.77 (0.68–0.83)	0.81 (0.74–0.91)	< 0.001

*Interquartile range, ^†^*p*-value from the Wilcoxon test for paired samples, ^‡^regions including central structures and all cerebral lobes.

**Figure 2:**
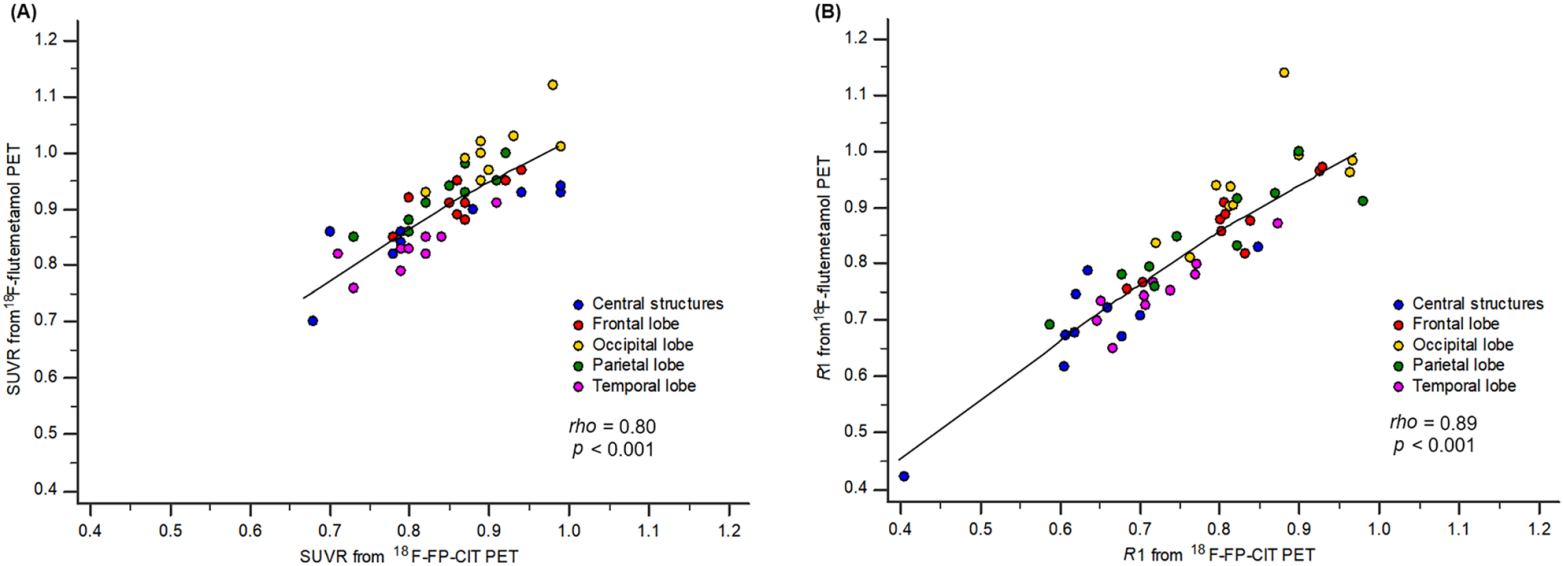
Scatter diagram of the correlation of parameters between ^18^F-FP-CIT PET and ^18^F-flutemetamol PET in the early phase. The early-phase SUVR obtained from ^18^F-FP-CIT PET showed a very strong correlation with that from ^18^F-flutemetamol PET in total brain regions including all cerebral lobes and central structures (*rho* = 0.80, *p* < 0.001, **A**). A very strong correlation of *R*1 was also observed between the two PETs in total brain area (*rho* = 0.89, *p* < 0.001, **B**). The trend line is drawn with the local weighted regression smoothing span (100%) in each diagram.

In total brain regions, the SUVRs obtained from ^18^F-FP-CIT PET showed a very strong correlation with those from ^18^F-flutemetamol PET (*rho* = 0.80, *p* < 0.001, Fig. 2A). There was a moderate degree of significant correlation of SUVRs from the two PETs in the frontal (*rho* = 0.69, *p* = 0.026), occipital (*rho* = 0.74, *p* = 0.014) and temporal lobes (*rho* = 0.78, *p* = 0.008), and very strong correlation in the central structures (*rho* = 0.85, *p* = 0.002) and parietal lobe (*rho* = 0.89, *p* < 0.001).

### Time-activity curves (TACs) from early-phase ^18^F-FP-CIT and ^18^F-flutemetamol PET scans

**Figure 3:**
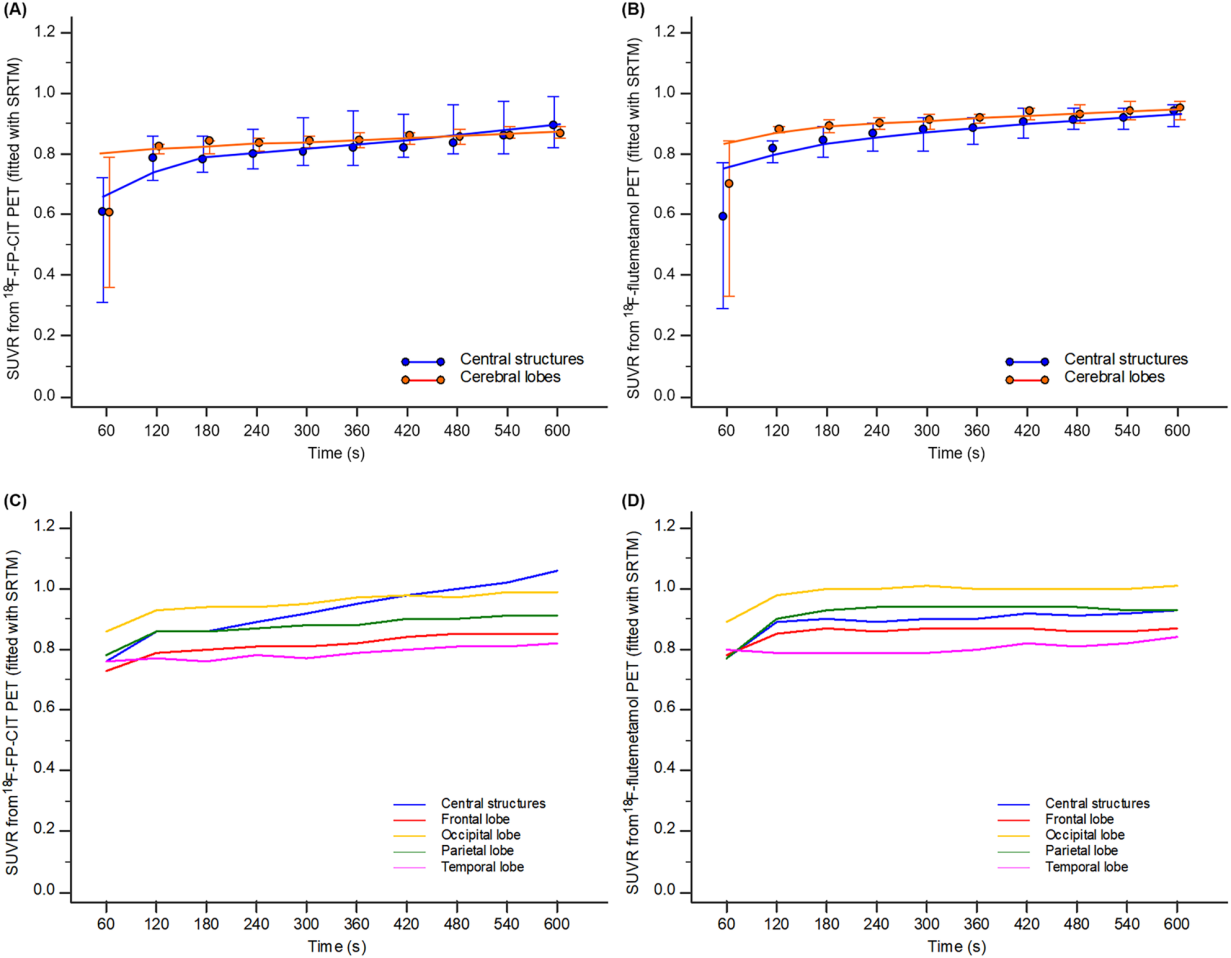
The SUVR time-activity curves (TACs) fitted using the simplified reference tissue method (SRTM) obtained from early-phase ^18^F-FP-CIT (**A**) and ^18^F-flutemetamol PET (**B**) images. The SUVR of ^18^F-FP-CIT in the central structure rose relatively steeply compared to other brain regions after 9 min (blue connecting line in **A**). Error bars represent the interquartile range of the median. Typical example of SUVR TACs using SRTM fitting in a patient (**C**,**D**).

The SUVR TACs from early-phase ^18^F-FP-CIT and ^18^F-flutemetamol PET fitted using a simplified reference tissue model (SRTM) are shown in Fig. 3. From 9 min onward, the ^18^F-FP-CIT SUVR of the central structures was higher than that of the cerebral lobes (Fig. 3A). However, this pattern was not observed until 10 min in the SUVR TACs from ^18^F-flutemetamol PET (Fig. 3B). Representative and typical SRTM fitting for SUVR TACs of a patient are shown in Fig. 3C,D. The individual SUVR TACs fitted using SRTM from 10 patients were provided in Supplementary Fig. 1.

### Kinetic parameters from early-phase ^18^F-FP-CIT and ^18^F-flutemetamol PET scans

The delivery rate of ^18^F-FP-CIT in total brain regions relative to the rate of delivery in the cerebellum (0.77 [0.68–0.83]), represented as *R*1, was significantly lower than that of ^18^F-flutemetamol (0.81 [0.74–0.91], *p* < 0.001). This significant difference in *R*1 between the two PETs was consistent across all brain areas even when dividing by each region (all *p* < 0.05, Table 1). There was a very strong correlation in *R*1 between ^18^F-FP-CIT and ^18^F-flutemetamol PET in total brain regions (*rho* = 0.89, *p* < 0.001, Fig. 2B). A very strong correlation of *R*1 between the two PETs was seen in the central structures (*rho* = 0.87, *p* = 0.001), frontal (*rho* = 0.91, *p* < 0.001), temporal (*rho* = 0.91, *p* < 0.001) and parietal lobes (*rho* = 0.88, *p* < 0.001). The occipital lobe demonstrated a moderate degree of significant correlation (*rho* = 0.65, *p* = 0.040). There were no significant differences or correlations in the efflux rate constant (*k*2) or binding potential (*BP*_*ND*_) between the two PETs for any brain regions (Supplementary Table 1).

## Discussion

We hypothesized at the beginning of this study that ^18^F-FP-CIT and ^18^F-flutemetamol activity in early-phase PET would be similar. However, comparing the two PETs showed that the cortical SUVR of ^18^F-FP-CIT was significantly lower than that of ^18^F-flutemetamol in the early phase, whereas there was no difference in SUVR in the central structures. To the best of our knowledge, no previous studies have compared early-phase PET using ^18^F-FP-CIT and ^18^F-flutemetamol. Therefore, it was difficult to find a precedent in the previous literature for the reasons underlying our results. The difference in SUVR between the two early-phase PET techniques is probably due to differences in their pharmacokinetic characteristics, and the apparently different shapes of TACs between the two PETs obtained in our study support this hypothesis. The SUVR of ^18^F-FP-CIT in the central structures does not differ from that of ^18^F-flutemetamol seems likely because of a steep increase in the activity of the central structures compared to other cortical regions on TACs. The central structures include the basal ganglia, the main target of ^18^F-FP-CIT, so this is not surprising. Although cortical SUVRs varied between the two PETs, they showed a moderate or very strong correlation in all brain regions.

Most previous studies that reported the usefulness of early-phase ^18^F-FP-CIT or amyloid PET performed validation with ^18^F-FDG PET^15,19–23^ or ^15^O-water perfusion PET^24^, but our study did not. Patients included in our retrospective study did not undergo ^18^F-FDG or perfusion PET imaging, so we cannot validate that early-phase PETs in our study reflect true brain perfusion. Further well-designed prospective studies including ^18^F-FDG PET or perfusion PET are needed to validate the current study. However, based on our results, we suggest that if both ^18^F-FP-CIT and ^18^F-flutemetamol PETs are scheduled within a short period of time, early-phase imaging from only one technique would be sufficient because of the significant correlation in their uptake. In addition, it may be helpful if clinicians keep in mind that ^18^F-FP-CIT may show lower uptake in cortical brain regions on early PET than ^18^F-flutemetamol.

Another notable result in our study is that *R*1 obtained from dynamic data demonstrated a significant correlation between the two PETs. The *R*1 also showed a significantly lower value in ^18^F-FP-CIT than in ^18^F-flutemetamol PET, similar to SUVR, but there was a very strong correlation for most brain regions. The *R*1, which represents the delivery rate of radiopharmaceuticals to the regional brain, has recently been used as a proxy for measuring cerebral blood flow in early-phase PET with ^18^F-flutemetamol^25^. In the central structures, SUVR showed no significant difference between the two PETs, but *R*1 was significantly different. It is difficult to clearly explain this discrepancy, but it is probably because the SUVR was obtained as the average value of the sum of the radiopharmaceutical activity over a 10 min duration, while the *R*1 value represents the delivery rate of radiopharmaceutical to the regional brain. On the other hand, *k*2 and *BP*_*ND*_ obtained failed to show any significant correlation between the two early PET techniques. It could be that our early-phase scan time of 10 min was not sufficient to estimate *k*2 and *BP*_*ND*_. In fact, Heeman et al.^26^ reported that a 60 min dual-time-window protocol of 0–30 and 90–110 min is needed to accurately estimate *BP*_*ND*_ in ^18^F-flutemetamol PET. Nevertheless, a strength of our study is that it demonstrated a significant correlation in the early phase of the two PETs with regard to the kinetic parameter *R*1 as well as SUVR. We would like to recommend pharmacokinetic modeling analysis in evaluating early phase PET images. Based on our results, it seems that it is necessary to evaluate early phase images to use pharmacokinetic modeling rather than simply to obtain SUVR.

In our study, ^18^F-flutemetamol was used as a radiopharmaceutical for amyloid PET. Previous studies that reported the usefulness of early-phase imaging with amyloid PET have used ^11^C-Pittsburgh Compound B^19,22,24,27,28^, ^18^F-florbetapir^20,23^, or ^18^F-florbetaben^15,18,28,29^, and we could find only single previous report using ^18^F-flutemetamol^26^. Since this study was carried out retrospectively, we could not select the radiopharmaceuticals used for amyloid PET. ^18^F-flutemetamol was simply the main radiopharmaceutical used in our institution, so this study dealt with ^18^F-flutemetamol. Thus, another strength of our study is that previous research reporting ^18^F-flutemetamol early-phase PET is very rare.

There is not yet a clear consensus on the optimal acquisition time for early-phase brain PET for ^18^F-FP-CIT and ^18^F-flutemetamol. Jin et al.^17^ conducted a study on the optimal time frame for ^18^F-FP-CIT early-phase PET, and reported that the 10 min image was the most useful, whereas the quality of the image was too poor at the 5 min or 7 min time points. Heeman et al.^26^ suggested the initial 30 min as the optimal time for early-phase ^18^F-flutemetamol PET imaging. At our institution, obtaining an initial 10 min image from both PETs is a routine protocol. Since our method has not been proven, this was an obvious limitation of this study. Therefore, further research to determine the image acquisition time that best reflects the brain perfusion status of each radiopharmaceutical is needed.

There are several limitations to this study and they are as follows. First, the number of subjects included in this study is small. The statistical sample size was indeed satisfied, but we admit that 10 subjects was small. Due to the cost burden, it was not easy to find patients who needed both ^18^F-FP-CIT PET and amyloid PET within the same month in our retrospective study. We look forward to future studies that will involve more subjects in order to validate our results. The second limitation was that we were unable to collect blood samples when acquiring dynamic images due to the retrospective research design. Therefore, we used SRTM, a kinetic model that can be used without blood sampling, which was also used in previous dynamic brain imaging studies^25,26,30^. In order to obtain results for other kinetic parameters that cannot be obtained from SRTM such as *k*1, future studies with blood sampling are warranted. The final limitation was that we could not enroll a homogeneous disease group. This study included patients with various diseases such as PD, PD with dementia (PDD), progressive supranuclear palsy (PSP), dementia with Lewy bodies (DLB), and AD. Although the disease groups varied, this should not present a major obstacle to comparing early uptake on PET performed at short intervals in the same patient, which was the goal of this study. However, studies in homogenous disease groups along with normal groups are needed to validate our results.

In conclusion, ^18^F-FP-CIT exhibited a lower level of cortical uptake than ^18^F-flutemetamol on early-phase PET, but uptake of both was significantly correlated.

## Methods

### Subjects

This study was conducted retrospectively. From September 2017 to September 2020, 15 patients were identified as having undergone both ^18^F-FP-CIT PET and ^18^F-flutemetamol PET from among the patient population at our single institution. All patients were clinically accompanied by cognitive impairment with parkinsonism symptoms, so both ^18^F-FP-CIT PET and ^18^F-flutemetamol PET were required. Of these, three patients who did not undergo early-phase PET imaging and two patients who did not have the magnetic resonance (MR) image data necessary for quantitative PET analysis were excluded. Finally, 10 patients (male/female = 6/4, median age 68 [IQR: 56–74] years, three patients with PD, three patients with PDD, two patients with PSP, one patient with DLB, and one patient with AD) were included. The interval between PETs for each patient was < 1 month (median 9 [IQR: 8–12] days). Also, MR images were acquired within 1 month of the PET images (median 6 [IQR: 5–11] days).

The clinical design of this retrospective study was approved by the Institutional Review Board of Ajou University (MED-MDB-20-511). The need for informed consent was waived.

### Brain PET/CT acquisition

PET/computed tomography (CT) data were acquired on a Discovery ST scanner (GE Healthcare, Milwaukee, WI, USA). All patients were forbidden to take neurology- or psychiatric-related drugs for 24 h before PET examination. The radiopharmaceuticals were purchased from commercial companies [^18^F-FP-CIT from DuChemBio (DuChemBi Co., Ltd., Seoul, South Korea) and ^18^F-flutemetamol from GE Healthcare (Vizamyl, GE Healthcare, Seoul, South Korea)]. Their radiochemical purity was confirmed and specific activity at the end of synthesis was sufficiently satisfactory to be used for PET imaging before daily use. For early-phase imaging, brain CT (100 kV, 95 mA; section width = 3.75 mm) was obtained, then 10 min dynamic PET data [60 s per frame, three-dimensional (3D) mode] were acquired immediately after intravenous injection of each radiopharmaceutical (median 201.83 [IQR: 191.66–207.20] MBq for ^18^F-FP-CIT and median 212.75 [IQR: 202.76–215.71] MBq for ^18^F-flutemetamol). Routine delayed-image acquisition was started 90 min after injection of radiopharmaceuticals. The delayed PET data [10 min per frame of 1 bed duration for ^18^F-FP-CIT and 20 min (4 × 5 min frames) for ^18^F-flutemetamol, 3D mode] were obtained after brain CT (same parameters as early phase). All PET images were iteratively reconstructed (i.e., ordered subsets of expectation maximization with two iterations and 21 subsets, Gaussian filter (full width at half maximum = 2.14 mm), with a 128 × 128 matrix) from CT data for attenuation correction.

### Quantitative analysis of early-phase PET images

All images were analyzed using Maximum Probability Atlas application in PMOD Neuro Tool (version 3.802, PMOD Technologies Ltd., Zurich, Switzerland). First, the averaged PET image was generated by averaging the frames from 0 to 10 min on the dynamic series. Then, the individual gray matter probability map was calculated by segmentation of each patient’s T1-weighted MR image. The brain was split into left and right hemispheres and the cerebellum. MR images were spatially normalized to the Montreal Neurological Institute (MNI) T1 template. The segmented and normalized MR images were rigidly matched to the averaged PET image, and their alignments were visually checked by a specialist in nuclear medicine with 13 years of brain PET experience (YS An). The automated anatomic labeling (AAL)-merged atlas^31^ was transformed to MR space and cortical structures were intersected with the gray matter probability map (mask threshold of 0.3). The final VOIs applied to the matched PET series for calculating average regional uptake, represented as the standardized uptake value (SUV), were based on body weight. The VOIs of central structures, frontal, occipital, parietal and temporal lobe regions were selected. Averaged SUVs from each brain region were divided by averaged cerebellar SUV to obtain SUVR, and SUVR images were generated based on the method published by Peretti et al.^32^.

**Figure 4:**
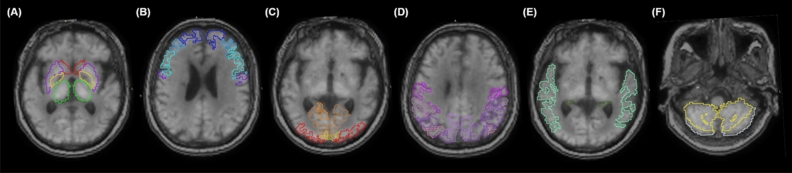
Representative images for outline contours of volumes of interest (VOIs). The VOIs for central structures (**A**), frontal (**B**), occipital (**C**), parietal (**D**), temporal (**E**) lobes, and cerebellum (**F**) with colored outline contours were automatically set in co-registered PET-MR images by the AAL-merged atlas provided by PMOD.

Also, the TAC of each region was obtained, and TACs were transferred to the kinetic modeling tool [PMOD Kinetic Modeling (PKIN)]. SRTM was developed with the cerebellum as a reference tissue. TACs fitted with SRTM and kinetic parameters including relative *R*1, *k*2, and *BP*_*ND*_ were obtained using a coupled fit across the VOIs^33^. The detailed structures constituting each brain area are shown in Table 2, and the representative outline contours of VOIs for selected areas are shown in Fig. 4.

**Table 2:** The structures included in each brain region.

Regions	Central structures	Frontal lobe	Occipital lobe	Parietal lobe	Temporal lobe	Cerebellum
Structures	Caudate nucleus	Precentral gyrus	Calcarine fissure and surrounding cortex	Postcentral gyrus	Temporal, superior, mid, inferior, poles	Vermis
	Putamen Pallidum	Rolandic operculum	Cuneus	Supramarginal gyrus	Amygdala	Cerebellum crus
	Thalamus	Supplementary motor area	Lingual gyrus	Angular gyrus	Hippocampus and parahippocampus	Cerebellum
		Olfactory cortex	Lateral remainder of occipital lobe	Precuneus	Fusiform gyrus	
		Superior frontal gyrus		Parietal, superior and inferior	Heschl’s gyrus	
		Middle frontal gyrus				
		Inferior frontal gyrus				
		Gyrus rectus				
		Paracentral lobule

### Statistical analysis

All statistical analyses were performed using MedCalc software (version 19.3.1; MedCalc Software bvba, Ostend, Belgium). Power analysis was used to calculate the sample size required for this study using a significance (α) level of 5% and statistical power (1 − β) of 80%. A sample size of five for paired samples *t* test and nine for correlation coefficient test was required to obtain an appropriate confidence level; thus, our final enrolled number of subjects (*n* = 10) satisfied these requirements.

Data in our study did not follow a normal distribution as assessed by the Kolmogorov–Smirnov test. Therefore, all continuous variables are presented as the median and IQR, and appropriate nonparametric statistical methods were used to analyze the data. The Wilcoxon test for paired samples was used to determine whether a difference existed between the parameters (i.e., SUVRs and kinetic parameters) obtained from ^18^F-FP-CIT and ^18^F-flutemetamol PET. The Spearman’s coefficient for the ranked correlation test was used to assess the correlation of parameters between ^18^F-FP-CIT and ^18^F-flutemetamol PET. The magnitude of the correlation was interpreted as poor (|*rho*| < 0.3), fair (|*rho*| = 0.30–0.59), moderate (|*rho*| = 0.60–0.79), or very strong (|*rho*| ≥ 0.80)^34^. A *p*-value of less than 0.05 was considered statistically significant.

## Ethics declarations

This retrospective study was conducted in accordance to the guidelines of the Declaration of Helsinki and approved by the Institutional Review Board of Ajou University (MED-MDB-20-511), through which informed consent was waived.

## Supplementary Information

Supplementary Figure 1.

Supplementary Table 1.

## Data Availability

The datasets used and/or analyzed during the current study are available from the corresponding author on reasonable request.

## References

[1] Mhyre TR, Boyd JT, Hamill RW, Maguire-Zeiss KA (2012). Parkinson's disease. Subcell Biochem..

[2] 2020 Alzheimer's disease facts and figures. *Alzheimers Dement*. 10.1002/alz.12068 (2020). 10.1002/alz.12068 32157811

[3] Yang Y, Cheon M, Kwak YT (2017). ^18^F-FP-CIT positron emission tomography for correlating motor and cognitive symptoms of Parkinson's disease. Dement. Neurocogn. Disord..

[4] Wang J (2007). ^18^F-FP-CIT PET imaging and SPM analysis of dopamine transporters in Parkinson's disease in various Hoehn & Yahr stages. J. Neurol..

[5] Yeo JM, Waddell B, Khan Z, Pal S (2015). A systematic review and meta-analysis of (18)F-labeled amyloid imaging in Alzheimer's disease. Alzheimers Dement. (Amst.).

[6] Song IU, Yoo I, Chung YA, Jeong J (2015). The value of brain perfusion SPECT for differentiation between mildly symptomatic idiopathic Parkinson's disease and the Parkinson variant of multiple system atrophy. Nucl. Med. Commun..

[7] Matsuda H (2007). Role of neuroimaging in Alzheimer's disease, with emphasis on brain perfusion SPECT. J. Nucl. Med..

[8] Borghammer P (2012). Cerebral oxygen metabolism in patients with early Parkinson's disease. J. Neurol. Sci..

[9] Rahmim A, Zaidi H (2008). PET versus SPECT: Strengths, limitations and challenges. Nucl. Med. Commun..

[10] Tatlidil R, Luther S, West A, Jadvar H, Kingman T (2000). Comparison of fluorine-18 deoxyglucose and O-15 water PET in temporal lobe epilepsy. Acta Neurol. Belg..

[11] Wong CY (2006). A statistical investigation of normal regional intra-subject heterogeneity of brain metabolism and perfusion by F-18 FDG and O-15 H_2_O PET imaging. BMC Nucl. Med..

[12] Teipel S (2015). Multimodal imaging in Alzheimer's disease: Validity and usefulness for early detection. Lancet Neurol..

[13] Jin S (2013). Differential diagnosis of parkinsonism using dual-phase F-18 FP-CIT PET imaging. Nucl. Med. Mol. Imaging.

[14] Hong CM, Ryu HS, Ahn BC (2018). Early perfusion and dopamine transporter imaging using (18)F-FP-CIT PET/CT in patients with parkinsonism. Am. J. Nucl. Med. Mol. Imaging.

[15] Son SH (2020). Early-phase ^18^F-florbetaben PET as an alternative modality for ^18^F-FDG PET. Clin. Nucl. Med..

[16] Segovia F (2018). Usefulness of dual-point amyloid PET scans in appropriate use criteria: A multicenter study. J. Alzheimers Dis..

[17] Jin S (2017). Additional value of early-phase ^18^F-FP-CIT PET image for differential diagnosis of atypical parkinsonism. Clin. Nucl. Med..

[18] Daerr S (2017). Evaluation of early-phase [(18)F]-florbetaben PET acquisition in clinical routine cases. Neuroimage Clin..

[19] Oliveira FPM (2018). Can ^11^C-PiB-PET relative delivery R1 or ^11^C-PiB-PET perfusion replace ^18^F-FDG-PET in the assessment of brain neurodegeneration?. J. Alzheimers Dis..

[20] Ottoy J (2019). (18)F-FDG PET, the early phases and the delivery rate of (18)F-AV45 PET as proxies of cerebral blood flow in Alzheimer's disease: Validation against (15)O-H_2_O PET. Alzheimers Dement..

[21] Peretti DE (2019). Relative cerebral flow from dynamic PIB scans as an alternative for FDG scans in Alzheimer's disease PET studies. PLoS One.

[22] Rostomian AH, Madison C, Rabinovici GD, Jagust WJ (2011). Early ^11^C-PIB frames and ^18^F-FDG PET measures are comparable: A study validated in a cohort of AD and FTLD patients. J. Nucl. Med..

[23] Hsiao IT (2012). Correlation of early-phase ^18^F-florbetapir (AV-45/Amyvid) PET images to FDG images: Preliminary studies. Eur. J. Nucl. Med. Mol. Imaging.

[24] Chen YJ (2015). Relative ^11^C-PiB delivery as a proxy of relative CBF: Quantitative evaluation using single-session ^15^O-water and ^11^C-PiB PET. J. Nucl. Med..

[25] Heeman F (2021). Simulating the effect of cerebral blood flow changes on regional quantification of [(18)F]flutemetamol and [(18)F]florbetaben studies. J. Cereb. Blood Flow Metab..

[26] Heeman F (2019). Optimized dual-time-window protocols for quantitative [(18)F]flutemetamol and [(18)F]florbetaben PET studies. EJNMMI Res..

[27] Rodriguez-Vieitez E (2017). Comparability of [(18)F]THK5317 and [(11)C]PIB blood flow proxy images with [(18)F]FDG positron emission tomography in Alzheimer's disease. J. Cereb. Blood Flow Metab..

[28] Tiepolt S (2016). Early [(18)F]florbetaben and [(11)C]PiB PET images are a surrogate biomarker of neuronal injury in Alzheimer's disease. Eur. J. Nucl. Med. Mol. Imaging.

[29] Martinez G (2017). 18F PET with florbetaben for the early diagnosis of Alzheimer's disease dementia and other dementias in people with mild cognitive impairment (MCI). Cochrane Database Syst. Rev..

[30] Yaqub M (2007). Quantification of dopamine transporter binding using [^18^F]FP-beta-CIT and positron emission tomography. J. Cereb. Blood Flow Metab..

[31] Tzourio-Mazoyer N (2002). Automated anatomical labeling of activations in SPM using a macroscopic anatomical parcellation of the MNI MRI single-subject brain. Neuroimage.

[32] Peretti DE (2019). Diagnostic performance of regional cerebral blood flow images derived from dynamic PIB scans in Alzheimer's disease. EJNMMI Res..

[33] Nelissen, N., Warwick, J., Dupont, P. & Leuven, K. U. Kinetic modelling in human brain imaging. *Positron Emission Tomography-Current Clinical and Research Aspects*, 978–953 (2012).

[34] Chan YH (2003). Biostatistics 104: Correlational analysis. Singapore Med. J..

