# Supplementary material for: Early-phase 18F-FP-CIT and 18F-flutemetamol PET were significantly correlated
Source: Sci Rep. 2021 Jun 10;11:12297. doi: 10.1038/s41598-021-91891-z (PMC8192502; doi:10.1038/s41598-021-91891-z)
Supplement: Supplementary file 1 — Supplementary Figure 1. [file 41598_2021_91891_MOESM1_ESM.doc]

Early-phase ^18^F-FP-CIT and ^18^F-flutemetamol PET were significantly correlated

Young-Sil An^1*^, Jung Han Yoon^2^, Sang Joon Son^3^, Chang Hyung Hong^3^, Su Jin Lee^1^, Joon-Kee Yoon^1^

*^1^Department of Nuclear Medicine and Molecular Imaging, ^2^Department of Neurology, ^3^Department of Psychiatry, Ajou University School of Medicine, Suwon, Korea*

^*^Corresponding author: Young-Sil An, Associate Professor

Address: Department of Nuclear Medicine and Molecular Imaging, School of Medicine, Ajou University, 206, World cup-ro, Yeongtong-gu, Suwon-si, Gyeonggi-do, Suwon, Korea 16499

Phone: +82-31-219-5947

Fax: +82-31-219-5950

E-mail: [aysays77@naver.com](mailto:aysays77@naver.com)

**
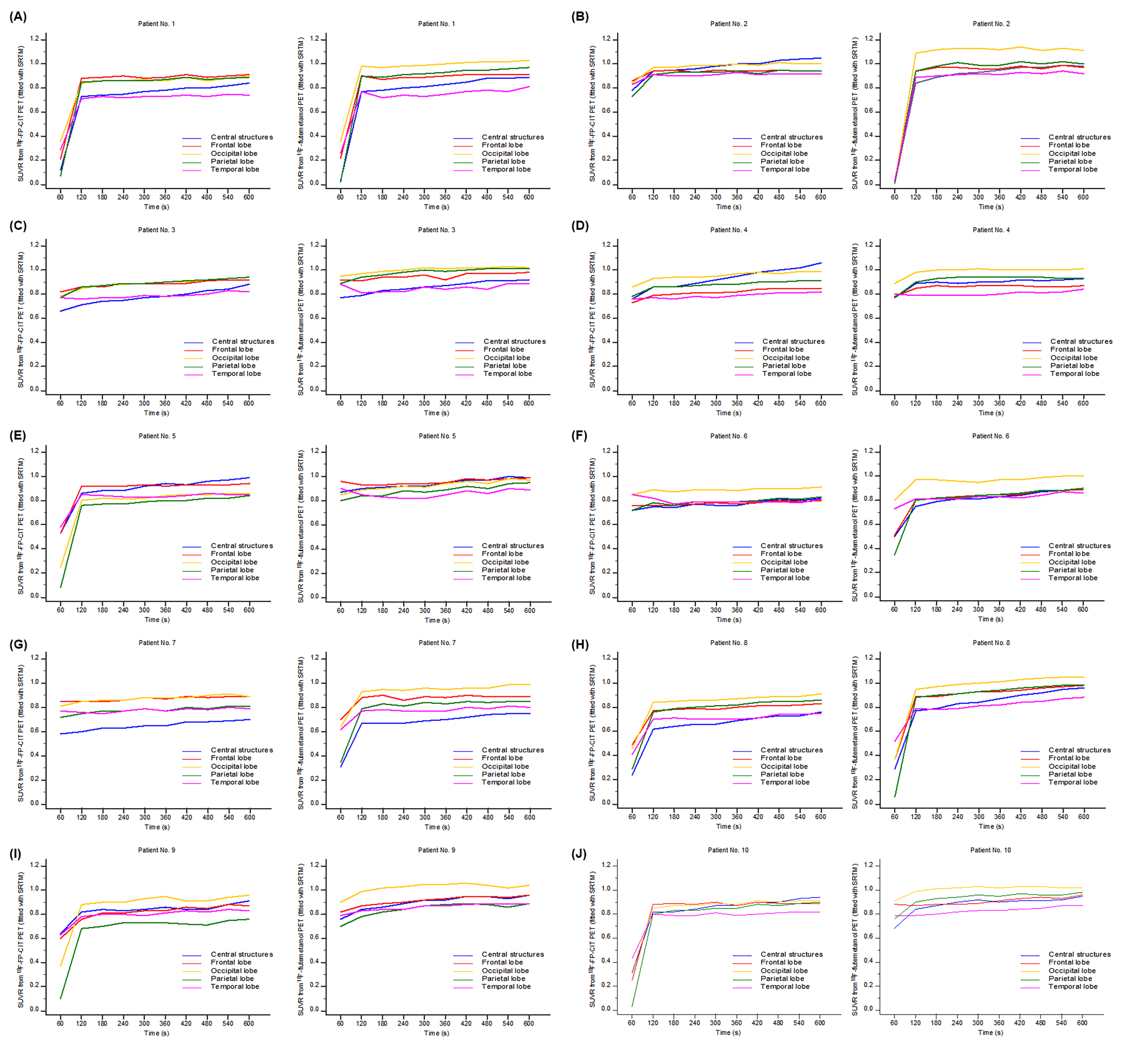
**

**Supplementary Figure 1.** Time-activity curves (TACs) fitted with SRTM of early-phase PETs in each patient. The SUVR TACs obtained from early-phase PET of ^18^F-FP-CIT is shown in the left graph and that from early-phase ^18^F-flutemetamol PET in the right graph in each of 10 patients (from A to J, patient number 1 to 10, respectively).
