# Supplementary material for: Early-phase 18F-FP-CIT and 18F-flutemetamol PET were significantly correlated
Source: Sci Rep. 2021 Jun 10;11:12297. doi: 10.1038/s41598-021-91891-z (PMC8192502; doi:10.1038/s41598-021-91891-z)
Supplement: Supplementary file 2 — Supplementary Table 1. [file 41598_2021_91891_MOESM2_ESM.doc]

Early-phase ^18^F-FP-CIT and ^18^F-flutemetamol PET were significantly correlated

Young-Sil An^1*^, Jung Han Yoon^2^, Sang Joon Son^3^, Chang Hyung Hong^3^, Su Jin Lee^1^, Joon-Kee Yoon^1^

*^1^Department of Nuclear Medicine and Molecular Imaging, ^2^Department of Neurology, ^3^Department of Psychiatry, Ajou University School of Medicine, Suwon, Korea*

^*^Corresponding author: Young-Sil An, Associate Professor

Address: Department of Nuclear Medicine and Molecular Imaging, School of Medicine, Ajou University, 206, World cup-ro, Yeongtong-gu, Suwon-si, Gyeonggi-do, Suwon, Korea 16499

Phone: +82-31-219-5947

Fax: +82-31-219-5950

E-mail: [aysays77@naver.com](mailto:aysays77@naver.com)

**Supplementary Table 1**. Comparison of kinetic parameters between ^18^F-FP-CIT PET and ^18^F-flutemetamol PET in the early phase.

|  | *k*2 (1/min) | | | |  | *BP_ND_* | | | |
| --- | --- | --- | --- | --- | --- | --- | --- | --- | --- |
|  | ^18^F-FP-CIT PET  (median, IQR^*^) | ^18^F-flutemetamol PET  (median, IQR^*^) | *p*-value^†^ for difference | *p*-value^‡^ for correlation (*rho)* |  | ^18^F-FP-CIT PET  (median, IQR^*^) | ^18^F-flutemetamol PET  (median, IQR^*^) | *p*-value^†^ for difference | *p*-value^‡^ for correlation (*rho)* |
| Central structures (*n* =10) | 0.04 (0.02–0.08) | 0.05 (0.01–0.06) | 0.997 | 0.556 (0.21) |  | 0.04 (0.00–19.99) | 0.07 (0.00–19.99) | 0.998 | 0.490 (0.25) |
| Frontal lobe (*n* =10) | 0.02 (0.01–0.04) | 0.03 (0.01–0.07) | 0.769 | 0.751 (0.12) |  | 0.00 (0.00–20.00) | 10.05 (0.00–20.00) | 0.164 | 0.351 (0.33) |
| Occipital lobe (*n* =10) | 0.07 (0.02–0.11) | 0.07 (0.01–0.65) | 0.375 | 0.702 (0.14) |  | 0.00 (0.00–0.16) | 0.38 (0.06–20.00) | 0.193 | 0.305 (0.36) |
| Parietal lobe (*n* =10) | 0.07 (0.02–0.08) | 0.09 (0.03–0.10) | 0.090 | 0.987 (0.01) |  | 0.00 (0.00–19.91) | 0.04 (0.00–0.35) | 0.547 | 0.878 (0.06) |
| Temporal lobe (*n* =10) | 0.01 (0.01–0.03) | 0.01 (0.01–0.02) | 0.557 | 0.855 (0.07) |  | 19.99 (0.00–20.00) | 20.00 (20.00–20.00) | 0.250 | 0.807 (0.09) |
| Total brain regions^§^ (*n* =50) | 0.03 (0.01–0.06) | 0.04 (0.01–0.09) | 0.060 | 0.122 (0.22) |  | 0.00 (0.00–20.00) | 0.24 (0.00–20.00) | 0.133 | 0.822 (0.03) |

^*^interquartile range, ^†^*p*-value from the Wilcoxon test for paired samples, ^‡^*p*-value from Spearman’s coefficient for the ranked correlation test, ^§^regions including central structures and all cerebral lobes
